# Semi-Annual Meeting of the Erie County Medical Society, June 10th, 1873

**Published:** 1873-07

**Authors:** D. E. Chace

**Affiliations:** Secretary


ART. II.—*Semi-Annual Meeting of the Erie County Medical Society, June* 10, 1873. Reported by D. E. Chace, M. D., Secretary.

The semi-annual meeting of the Erie County Medical Society convened at the rooms of the Buffalo Medical Association in the Y. M. A. buildings at half-past ten o'clock Tuesday, June 10th. Dr. Jabez Allen, the President, upon taking the chair, made a few remarks upon the large attendance, and of the importance, especially to the younger members of the society, of these meetings.

The Secretary presented the diplomas of Drs. D. C. Hunter, John Dambach, Geo. W. McPherson, Joseph Fowler, Alfred T. Livingston, I. Brooks and Daniel A. Bailey, with their applications for membership, which were referred to a committee composed of Drs. T. M. Johnson, Shaw and Ring, who reported in favor of their admission to membership upon compliance with the by-laws.

Dr. J. F. MINER moved a suspension of the usual order of business to introduce Dr. Levi Ham, of Indiana, a former member of the society.

Dr. HAM being invited to address the members present, acknowledged the courtesy extended to him, and in a brief but pleasing effort, recalled the names of those who some twenty years since were the active leading spirits of the medical profession, men whose names he would mention only to stimulate those who have succeeded them to the practice of a noble profession with so marked degree of success, and whose lives were noted for their sincerity of purpose and disinterestedness, who had left behind them as monuments their honorable records engraven in the hearts of their numerous patients, and fully appreciated by the public. He was glad to see so many young men entering upon the practice of medicine, and their presence here to-day was an evidence of the interest felt by them towards their profession and its welfare.

A vote of thanks was tendered to Dr. Ham for his address.

Dr. E. R. BARNES was nominated by the committee as orator, and Dr. M. B. Folwell as alternate for the next annual meeting in January, 1874.

Dr. SAMO, the Librarian, read a letter from Dr. C. E. Brown-Sequard, tendering the society a copy of the "Archives of Scientific and Practical Medicine." The communication was received with thanks.

Dr. T. M. JOHNSON moved that a committee of three be appointed to arrange an amendment to the by-laws for the purpose of having monthly meetings of the Society, and also to try and procure a suitable room in this building to be used exclusively for medical purposes.

Dr. WM. GOULD, delegate to the State Medical Society, read the following report of the proceedings of that body at its annual meeting in February:

The undersigned, delegates from this society to the State

Medical Society, beg leave, respectfully, to report that we attended the session of the State Society, which met in the hall in the Perry Building, City of Albany, Feb. 4th, 1873.

At the usual hour President Agnew, of New York, called the meeting to order and delivered his inaugural address, after which the following committees were announced, viz.:

ON CREDENTIALS.—Drs. W. H. Craig, of Albany; A. L. Saunders, of Madison; and C. E. Rider, of Rochester.

BUSINESS COMMITTEE.—Drs. Ellsworth Elliott, New York; W. Kendall, Baldwinsville; G. H. Hubbard, Lansingburg.

COMMITTEE ON ARRANGEMENTS AND RECEPTIONS.—Drs. Quackenbush and Bailey, Albany; and Dr. H. Colvin, of Wayne County.

COMMITTEE ON MEDICAL ETHICS.—Dr. Thomas Hun, of Albany; Dr. E. R. Squibb, Brooklyn; and Dr. D. B. St. John Roosa, New York.

On motion a committee consisting of Drs. Fraser, of Oneida; Lewis Post, of Seneca; and Thompson Burton, of Montgomery, were appointed to invite the Governor and such members of the Legislature as belonged to the regular profession, to be present at the sessions of the Society. Whether there were any such members of the Legislature present we cannot say, but we are quite sure that the form of our venerable Governor was at no time visible in the hall.

Papers on the following subjects were presented, viz., "A case of Occlusion of the Femoral Artery from fracture of the femur, followed by mortification and amputation," by Dr. George Burr, of Binghampton. "Disease of the left Ovary, resulting in fatal Hæmorrhage," by Dr. E. H. Bridges, of Ogdensburg. "Obituary notice of Dr. Darius Clark," by Dr. B. F. Sherman, V. P. "On Hernia," by Dr. J. H. Pooley, of Yonkers. "The effect of Rail Road travel on the health of Women," by Dr. Eli Vanderwarker. "Idiopathic Peritonitis," by Dr. Lewi, of Albany. All of which was referred to the publishing committee.

Dr. D. B. St. John Roosa, of New York, read a paper entitled "History of the progress of Otology," which was discussed in a very able manner by Dr. Knapp, of New York, and then referred to the committee on publication. Dr. W. B. Alley, of Nunda, read a paper on "The ultimate result of nerve injuries in gun shot wounds," which was discussed by several members. At this point the business committee made enquiry of the President as to what disposition was to be made of papers presented by invited members, and was instructed to cause them to be read if they deemed it expedient.

The following committee on the President's address was announced; Drs. Vanderpoel, Gray, and Krackowizer.

An interesting paper was then read on "Perineal Lithotomy," by Dr. J. W. S. Gouley, of New York.

The President announced the committee on nominations: 1st District, Dr. J. C. Hutchinson; 2nd District, Dr. J. F. Jenkins; 3d District, Dr. H. B. Whiton; 4th District, Dr. Alexander Ayres; 5th District, Dr. Alonzo Churchill; 6th District, Dr. William C. Wey; 7th District, Dr. Caleb Green; 8th District, Dr. H. W. Dean.

It will be borne in mind that this is a very important committee. It is their duty to nominate officers of the society for the ensuing year, consisting of a President, a Vice-President, a Secretary, and Treasurer. Three Censors for each of the four districts into which the State is divided; and also to select two candidates for each of the eight senatorial districts from those eligible for permanent membership. We say *for* each district instead of *from* for the reason that candidates are sometimes selected from other than their own districts, a circumstance which has led to some remonstrance from the slighted districts. Each district is entitled to two permanent members annually, and, gentlemen, if any of you should aspire to the honors of office or permanent membership, you must lay your pipe and do your log-rolling with this committee, as I believe their selections are always approved and their nominations confirmed by the society. We cannot say that w like the system for it enables a few master-spirits to control the whole matter, and that result is almost sure to follow the permanent location of any society, comprising a State jurisdiction as large as that of New York. But to return from this digression.

Dr. Thomas Addis Emmet read a paper on "Laceration of the Perineum, involving the Sphincter and Operation for securing union of the Muscle."

Dr. E. H. Parker read a paper on "Dislocation of the Tarsus from the Astrugalus."

Dr. Moore, of Rochester, presented and read a paper on "Intra Capsular Fracture," with illustrations, on which a debate sprang up, participated in by Drs. Gurdon, Buck, Bell, Douglas and others. Several other papers were presented and referred without reading to the committee on publication. The report of the committee on changing the time of meeting of the society was taken from the table and discussed pro and con until the hour of adjournment. The above comprises the main points of the first days' proceedings.

Second day.—At 9 o'clock the President called the society to order. The minutes of yesterday's proceedings were read and adopted. The committee on By-Laws made a report, which was received. A paper on "Strictures of the Male Urethra, with results of Operation, with the Dilating Urethrotome," was read by Dr. F. W. Otis, of New York, and discussed by Drs. Gouley and Newman, of New York. At this point Dr. Roosa, of New York, rose to a question of privilege, denying or repudiating a statement made last year by Dr. Stephen Rogers. If we recollect, the statement charged certain of the New York Colleges with selling cheap Diplomas, which Dr. Roosa emphatically denied.

Dr. Elliott offered the following resolution:

*Resolved* that candidates, elected to permanent membership, who neglect to pay the fee, required by our By-Laws for one year from the date of their election, shall forfeit their rights as permanent members. *Adopted.*

Dr. A. N. Bell made a report on Quarantine Establishment in New York, and Dr. A. B. Burger presented a specimen of diseased kidney, with a history of the case. About half a paper on the Pathology of Labor Pains was read by Dr. W. T. Lusk, of New York, and the reason we did not hear the other half was that the time allowed by the business committee was not sufficient for the purpose, and the time not extended, a courtesy granted or denied as suited the temper of the society. Whether in this case the paper was not of sufficient interest or its author under a cloud we cannot say, but the reading was interrupted very unceremoniously when the ten minutes expired, notwithstanding the Doctor's apparent discomfiture caused by the Autocrat, at the head of the business committee, calling "Time!" "Time." Those who have not been there may not be aware how the business of the society is managed by this "Business committee," and if you have the patience, perhaps we might as well explain it a little at this point.

Any one desiring to present a paper goes to the "Business committee, and if they approve it they allow so many minutes, five, ten or fifteen as they please, and call for its reading when it suits their convenience, and when the time allotted expires the reader subsides, whether half through or not, unless the time is extended by a vote of the society, a rule which may be right, but to a Neophyte it looks a little arbitrary to say the least.

Dr. Gurdon Buck, a veteran in the profession, next presented a case of reconstruction of the under lip, accompanied with models and explanations.

Dr. Lewis A. Sayre read a paper on "Diastasis of Head, of Femur, and formation of Artificial Hip joint," both of which were referred to the publishing committee.

Dr. Quackenbush, of Albany, read an interesting paper on "Hydrorrhœa," giving history of cases.

Next came the report of Dr. E. R. Squibb, one of the committee on Ethics, in regard to the difficulties in the Niagara County Medical Society. There were two sets of delegates from that county, each claiming to be the "Simon pure" representatives of the County Society.

As many present may not understand what the trouble is in that county, if not tresspassing too much on your time and patience, we will explain it as well as we can.

The Medical Society of that county was organized some fifty years ago, more or less, and continued until the winter of 1873, when by a vote of the members present it was disbanded, and a new County Society at once organized, by which action about one-half of the former members were excluded on the ground of violations of the code of Ethics, Both parties keep up their organization and claim to be the true Medical Society. Two societies could not be recognized in the same county; hence the fight in the State Medical Society for recognition by the two sets of delegates. The case was referred to the committee on Ethics, and their report was in favor of the new organization, which left the old fogies and advertising adventurers out in the cold, and just what the new organization intended to do from the first. Whatever justice there may be in the case, there are grave doubts as to the legality of their action. The excluded members retain the books and property of the original society and keep up their organization, but their delegates are rejected by the State Society. It may be that the end is not yet. We are inclined to believe that if the members, claiming to be the original organization, are guilty of the irregularities charged by the new, they deserved their fate.

After some routine business Dr. Kendall offered a resolution altering the By-Laws so that section eleven of paragraph three should read as follows, viz.:

At the Annual Meeting, at the close of the morning session of the first day, the members of the society shall be organized into eight committees by senatorial districts as established by the law of 1836; the members present from each district constituting one committee, each of which shall elect one member. The members thus elected with one appointed by the President as Chairman shall constitute the committee of nominations.

This was laid upon the table temporarily, and we think the bed was so hard that it died, for it has not been heard of since.

This proposition if adopted would in a measure curtail the power of the President to select his successor. As it now stands he selects the nominating committee, who not only name the officers but also censors and two permanent members from each of the eight districts. Their selection we suppose is always approved as a matter of course by the society; the victors rejoicing over their success, and the vanquished chewing the cud of disappointment and vexation in silence.

The resolution offered by Dr. Storck in relation to the act passed by the Assembly and Senate in 1872 and vetoed by Governor Hoffman, was after a short discussion laid quietly to sleep on the table, and as near as we could judge of the temper of the society it will not be disturbed in our day and generation. A great drawback to the prosperity of the society, from what we gleaned from the discussion, is the time of meetings, first Tuesday in February, a time when Albany Hotels are full to overflowing and traveling from many portions of the State is both difficult and dangerous. To obviate these difficulties a resolution changing the time of meeting to the fourth Tuesday in September was offered, which led to a long discussion and to which many amendments were offered, but no satisfactory conclusion could be reached; the whole subject was laid upon the table, and so this much needed change ended in smoke, or perhaps more appropriately, in a huge volume of Gas. Following this, a number of papers on various subjects were presented, some of which were read and others not, all were referred to the publishing committee and in due time all or a portion only will be approved and published.

It appears from the report of the Censors that but one applicant received a Diploma during the year, and that was in the Eastern District. The proceedings of the second day closed with a reception given to the State Society by the Albany County Medical Society, which was not only a feast but a very pleasant social gathering.

Third day.—After the reading and adoption of the minutes, reports of committees and presentation of papers were resumed.

Dr. J. Marion Sims read a paper on "Enucleation and removal of an Intra Uterine Fibroid Tumor," and exhibited the instruments used in the operation.

Dr. J. N. Northrop, of Albany, presented a case of Congenital loss of the right arm. He claimed that the boy's arm was amputated in utero. His father was wounded in the corresponding arm, and he further claimed that the stump of the boy's arm showed the mark of a bullet wound or the imitation of one. His theory was demolished, fair as it looked at first sight, by Dr. Jacobi, of New York, who explained the phenomenon as a case of arrested development and that the bullet mark was only an undeveloped finger.

By resolution, the committee on Hygiene is hereafter to be recognized as one of the standing committees, and directed to place itself in immediate correspondence with the county societies. Also resolved that the standing committee on Hygiene consist of seven members, one of whom shall be Dr. C. R. Agnew, the remainder of the committee to be appointed by the chair. The President appointed as standing committee on Hygiene the following; Drs. Bell, Vanderpoel, Didama, Dean, Ordronaux, Smith and Agnew.

The nominating committee made the following report; for President, Dr. E. M. Moore, of Rochester. Vice President, Dr. Francis Burdick, of Johnstown. Secretary, Dr. W. H. Bailey, of Albany. Treasurer, Dr. C. H. Porter, of Albany.

For Censors, *Southern District,* Dr. E. R. Squibb, of Brooklyn; Dr. E. H. Parker, of Poughkeepsie; and Dr. Ellsworth Elliot, of New York.

*Eastern District,* Dr. John P. Sharer, of Hirkimer; Dr. J. L. Babcock, of Albany; Dr. G. H. Hubbard, of Lansingburg.

*Middle District,* Dr. M. M. Bagg, of Utica; Dr. Horace Lathrop, of Cooperstown; Dr. C. G. Bacon, of Fulton.

*Western District,* Dr. Caleb Green, of Homer; Dr. C. C. Wyckoff, of Buffalo; Dr. D. Colvin, of Clyde.

*Permanent Members.*

1st District—Dr. Robert Newman, Dr. J. Marion Sims.

2nd District—Dr. T. B. Smith, Dr. W. H. Helm.

3rd District—Dr. E. R. Hun, Dr. R. H. Ward.

4th District—Dr. John Parr, Dr. D. G. Dodge.

5th District—Dr. H. G. P. Spencer, Dr. W. L. Baldwin.

6th District—Dr. J. H. Dolson, Dr. William Fitch.

7th District—Dr. G. W. Earl, Dr. E W. Simmons.

8th District—Dr. C. N. Palmer, Niagara County, Dr. J. R. Coates, Genesee County.

All of whom were unanimously elected as a matter of course, as it would require some nerve to oppose any of the gentlemen selected by the committee.

Dr. Jenkins offered a resolution, as a part of the report, that no one should be eligible for election to permanent membership until he had been a delegate for three years.

Dr. Squibb moved, as an amendment, that delegates should be present and serve for three years.

The amendment prevailed, and the resolution, as amended, was adopted. Hereafter the honors will have to be fairly earned by three years of actual attendance, at an expense of about one hundred dollars and loss of time for the delegates from this county.

There being no further business the President, after a few remarks appropriate to the occasion, declared the annual session closed.

Speaking of the obituary notice of the death of Doctor Darius Clark, by Dr. Sherman, reminds us of one of the most touching incidents noticed in the proceedings, and that is the manner in which the memory of deceased members of the society are recorded in the proceedings, and thus placed in the archives of the society, will be handed down to future generations. It was also a noticeable fact that all the medical gentlemen present were simply doctors. Every gentleman addressed was plain "Dr. A, B or C." High sounding titles were entirely ignored, which is certainly an evidence of good taste.

In conclusion, let me say that the business of the society, in all its workings, might be changed from the ancient regime to one better adapted to the present age. In the first place it should be made a moveable body; secondly, the officers should be elected by a ballot cast by the delegates present, and the members should be allowed to present and read papers the same as in any other popular assembly; and last, though not least, the time of meeting should be changed from February to some less inclement season. It is to be hoped that all these changes will be made.

Respectfully submitted,

W. Gould,<br>Jabez Allen, } Delegates.

Dr. Samo announced to the Society that he had been tendered by Dr. Levi Ham, for the use of the Society, a copy of the "Medical and Surgical History of the late War," prepared under the direction of Surgeon-General Barnes. The gift was accepted with the thanks of the Society for the complimentary donation. The usual routine business matters were then transacted and the Society adjourned.

